# Assessment of veins in T2*-weighted MR angiography predicts infarct growth in hyperacute ischemic stroke

**DOI:** 10.1371/journal.pone.0195554

**Published:** 2018-04-04

**Authors:** Susumu Yamaguchi, Nobutaka Horie, Minoru Morikawa, Yohei Tateishi, Takeshi Hiu, Yoichi Morofuji, Tsuyoshi Izumo, Kentaro Hayashi, Takayuki Matsuo

**Affiliations:** 1 Department of Neurosurgery, Graduate School of Biomedical Sciences, Nagasaki University, Nagasaki, Japan; 2 Department of Radiological Sciences, Graduate School of Biomedical Sciences, Nagasaki University, Nagasaki, Japan; 3 Department of Neurology and Strokology, Graduate School of Biomedical Sciences, Nagasaki University, Nagasaki, Japan; Hungarian Academy of Sciences, HUNGARY

## Abstract

**Background and purpose:**

T2*-weighted magnetic resonance angiography (SWAN) detects hemodynamic insufficiency as hypointense areas in medullary or cortical veins. We therefore investigated whether SWAN can help predict ischemic penumbra-like lesions in patients with acute ischemic stroke (AIS).

**Materials and methods:**

Magnetic resonance imaging (MRI) records—including SWAN, diffusion-weighted imaging (DWI), and magnetic resonance angiography (MRA)—of consecutive patients with major vessel occlusion within 6 h from AIS onset were analyzed. Acute recanalization was defined as an arterial occlusive lesion score of 2–3. A modified Alberta Stroke Program Early CT Score (mASPECTS) was used to evaluate ischemic areas revealed by SWAN and DWI. SWAN- and DWI-based mASPECTSs were calculated, and correlations between DWI-SWAN mismatches with final infarct lesions or clinical outcomes were evaluated.

**Results:**

Among the 35 patients included in this study, we confirmed cardioembolic stroke in 26, atherothrombotic stroke in 4, and unknown stroke etiology in 5. Overall, recanalization was achieved in 23 patients, who showed a higher follow-up DWI-based mASPECTS and lower modified Rankin Scale (mRS) score at 90 days than patients without recanalization. Initial SWAN- and follow-up DWI-based mASPECTSs were significantly higher for atherothrombotic stroke than for cardioembolic stroke. Of 12 patients without recanalization, DWI-SWAN mismatch was significantly correlated with infarct growth. Patients with recanalization showed no such correlation. In the assessment of clinical outcome, follow-up DWI-based mASPECTS and patient's age were significantly correlated with mRS at 90 days after stroke. A multivariate logistic regression analysis revealed that the follow-up DWI-based mASPECTS was independently associated with a favorable outcome 90 days after stroke.

**Conclusions:**

For patients with AIS, DWI-SWAN mismatch might show penumbra-like lesions that would predict infarct growth without acute recanalization. Assessment of ischemic lesions from the venous side appears to be useful for considering the etiology and revascularization therapy.

## Introduction

Treatment for patients with acute ischemic stroke (AIS) has recently made remarkable progress with the development of endovascular devices. Among these devices, stent retrievers have been able to achieve rapid recanalization and a high recanalization rate, thereby providing favorable outcomes for patients with AIS and major artery occlusion [1]. Endovascular thrombectomy up to 7.3 h after symptom onset is effective in patients with AIS [2]. Mechanical thrombectomy for patients with AIS who were known to be well 6–24 h earlier and had a mismatch between clinical deficits and infarction was reported to improve patients’ outcome in the DAWN trial. [3] The DEFUSE 3 study showed significant benefits of mechanical thrombectomy compared with medical therapy alone for patients with AIS 6–16 h after stroke onset [4]. These results could widen the treatment indications for AIS.

When examining an AIS patient, it is critical to evaluate the ischemic penumbra as rapidly as possible. Current methods for ischemic penumbra evaluation include positron emission tomography (PET), single photon emission computed tomography, and contrast-enhanced perfusion imaging by computed tomography (CT) or magnetic resonance imaging (MRI). These methods, however, are costly, and the use of contrast medium sometimes poses risks of allergy and renal impairment. Although non-contrast MRI takes time compared with CT perfusion, MRI could provide hemodynamic information by adding arterial spin labeling and susceptibility-weighted imaging (SWI) [5, 6].

T2*-weighted MR angiography (SWAN) is an imaging sequence term derived from GE Healthcare, and SWI is a term from Siemens. SWAN can also detect slight changes in susceptibility caused by microbleeding. Some authors have reported that a cortical vessel hypointensity sign on SWI or T2*-weighted imaging (T2*WI) in 3.0-T MRI can quickly and conveniently predict ischemic penumbra without contrast media [6–8]. We expect SWAN in 1.5-T MRI to detect hemodynamic insufficiency (which appears as hypointense areas in the medullary or cortical veins) as well as SWI or T2*WI in 3.0-T MRI. Some authors reported that hypointense cerebral vessels seen on SWI within 24 or 72 h from symptom onset predicted infarct growth and a poor outcome [9, 10]. No reports, however, have shown the prognosis of lesions or clinical outcomes, with or without recanalization, for major artery occlusion during the hyperacute phase, especially within 6 h from symptom onset.

In this study, we investigated (1) whether there are any differences in SWAN-based or diffusion-weighted imaging (DWI)-based mASPECTS between the initial and follow-up MRI results regarding recanalization/no recanalization and atherothrombotic versus cardiogenic stroke; (2) whether a DWI-SWAN mismatch could predict infarct growth with or without recanalization; and (3) which variables affect clinical outcome.

## Materials and methods

### Ethical standards

All studies on humans described in this manuscript were carried out in accordance with national law and the Helsinki Declaration from 1964 (in its present revised form). The data were analyzed anonymously in this study.

### Patients and clinical protocol

We retrospectively analyzed patients with AIS at our hospital from January 2012 to December 2014. Patients who showed acute major artery occlusion within 6 h from AIS onset were examined with MRI, including SWAN, DWI, and magnetic resonance angiography (MRA). We analyzed MRI at two stages: initial MRI (conducted at hospital admission) and follow-up MRI (conducted >24 h after AIS onset). Patients suffering from moyamoya disease or arterial dissection were excluded, as were patients in whom MRI or MRA assessments could not clearly determine acute recanalization within a few hours after initial treatment. The pathology in this study included occlusion of the internal carotid artery and or the M1 portion of the middle cerebral artery. Patients with other arterial occlusions were excluded.

**Fig 1:**
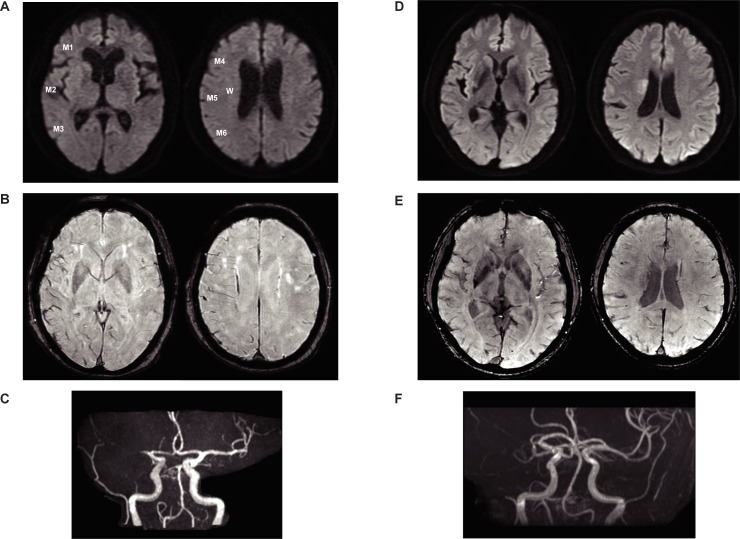
Representative case. Case 1, cardioembolic stroke. (**A**) Initial diffusion-weighted imaging (DWI)-based modified Alberta Stroke Program Early CT Score (mASPECTS) was 6 points. (**B**) Initial T2*-weighted MR angiography (SWAN)-based mASPECTS was 0 points. (**C**) Magnetic resonance angiography (MRA) shows right M1 occlusion. (**D**) Case 2, atherothrombotic stroke. (**E**) Initial DWI-based mASPECTS was 6 points. (E) Initial SWAN-based mASPECTS was 5 points. (**F**) MRA shows right M1 occlusion.

To evaluate ischemic areas using SWAN and DWI, a modified Alberta Stroke Program Early CT Score (mASPECTS) was defined as follows: A total of 7 points were obtained for each of the M1, M2, M3, M4, M5, M6, and W portions of the vessel (Fig 1) [11, 12]. When the totals for the cortical and medullary veins on the occluded side were higher than on the normal side, we defined it as abnormal *hypointensity* in the SWAN evaluation. *Hyperintensity* for DWI was defined as abnormal intensity. When the occluded area showed abnormal intensity, a value of “1” was subtracted from 7 (a perfect mASPECTS). After calculating SWAN- and DWI-based mASPECTSs, we calculated the DWI-SWAN mismatch as subtraction of the initial SWAN-based mASPECTS from the initial DWI-based mASPECTS. We then calculated infarct growth by subtracting the follow-up DWI-based mASPECTS from the initial DWI-based mASPECTS. One neurosurgeon and one neuroradiologist independently assessed the mASPECTSs for DWI and SWAN from MRI scans. When their decisions were discordant, another neurosurgeon made the final decision. Each observer judged the MRI findings in each area, and the inter-rater agreement was calculated. We defined the arterial occlusive lesion (AOL) score of 2–3 as recanalization because collateral flow could affect the fate of the tissue in the recanalization group. The AOL score was defined using MRA source images or digital subtraction angiography. In patients without digital subtraction angiography, MRA was performed approximately 1 h after initial treatment to assess acute recanalization.

A favorable outcome was defined as grades 0–2 by the modified Rankin Scale (mRS). The correlations between the DWI-SWAN mismatch and the final infarct lesion or the variables that affect clinical outcome were also evaluated.

### MRI protocol

All patients underwent MRI studies on a 1.5-T MR system (Signa HDx; GE Healthcare, Piscataway, NJ, USA) with an eight-channel head coil. The imaging sequence and parameters were as follows: For SWAN: repetition time (TR) 63.7 ms, effective echo time (TE) 49.8 ms, flip angle 15°, slice thickness 3 mm, field of view (FOV) 22 cm, matrix size 192 × 384, number of echoes 5, number of excitations (NEX) 0.69, total acquisition time 2 min 52 s. For DWI: TR 10000 ms, TE 81 ms, b value 1000 s/m^2^, slice thickness 5 mm, FOV 27 cm, matrix size 128 × 192, number of echoes 1, NEX 1, total acquisition time 50 s).

### Statistical analysis

Data were presented as medians (interquartile range: 25–75th percentiles). The significance of intergroup differences was assessed using Fisher’s exact test for categorical variables and the Mann–Whitney U test for continuous variables. The consistency of individual observations was evaluated by kappa (κ) statistics [13]. Spearman's rank correlation test was used to evaluate relations between variables across the groups. Statistical significance was defined as *P* < 0.05. No corrections were made for multiple comparisons because comparisons of mASPECTS were interpreted as exploratory analyses.

## Results

In all, 35 patients (median age 77 years, 16 men) met the criteria of our study. Cardioembolic stroke was observed in 26 patients and atherothrombotic stroke in 4. The other stroke etiologies were unknown. ICA occlusion was found in 13 patients and M1 occlusion in 22 patients. The median in-hospital National Institutes of Health Stroke Scale score was 16, and median times from onset to emergent destination and from onset to initial MRI were 89.0 min and 56.0 min, respectively. Intravenous tissue plasminogen activator was administered in 22 patients, and endovascular therapy was performed in 19 patients: mechanical thrombectomy in 14 patients, intra-arterial thrombolysis in 4 patients, and percutaneous transluminal angioplasty in 1 patient. Overall, recanalization was achieved in 23 (65.7%) patients.

Basic characteristics between the recanalization and no recanalization groups are shown in Table 1.

**Table 1:** Baseline characteristics for the recanalization and no recanalization groups.

	Recanalization (n = 23)	No recanalization (n = 12)	*P* value
**Age median (IQR)**	70.0 (60.0–82.0)	82.0 (71.3–87.0)	0.091
**Sex (male), n**	11	5	1.0
**Cardioembolic stroke, n**	18	8	0.65
**Occlusion site, n**	ICA 6, M1 17	ICA 7, M1 5	0.079
**Hypertension, n**	17	9	1.0
**Diabetes Mellitus, n**	3	1	1.0
**Hyperlipidemia, n**	9	1	0.11
**Current Smoke, n**	3	2	1.0
**OTE time, min, median (IQR)**	56.0 (41.0–109.0)	61.5 (22.5–93.8)	0.55
**OTM time, min, median (IQR)**	82.0 (65.0–127.0)	89.5 (67.5–177.5)	0.99
**Follow up MRI, hour, median (IQR)**	23 (18.5–24.5)	24.4 (21–106.4)	0.19
**In-hospital NIHSS median (IQR)**	14 (11.0–19.0)	20 (16.0–20.8)	0.085
**tPA, n**	15	7	0.73
**Endovascular treatment, n**	16	3	0.012
**OTR time, min, median (IQR)**	266 (184.0–337.0)	-	-

*P* values were calculated using the Mann–Whitney U test for comparing median values and by Fisher’s exact test for comparing relative proportions. IQR, interquartile range; ICA, internal carotid artery; MCA, middle cerebral artery; MRI, magnetic resonance imaging; NIHSS, National Institutes of Health stroke scale; tPA, tissue plasminogen activator; OTE, onset-to-emergent destination; OTM, onset-to-initial MRI; OTR, onset-to-recanalization.

The proportion of patients who underwent endovascular treatment in the recanalization group was significantly larger than that in the no recanalization group (*P* = 0.012) (Table 1). There were no significant differences for any of the other patients’ characteristics between the groups (Table 1). This evaluation by two observers showed moderate inter-rater agreement (κ = 0.54).

### Assessment of imaging

**Fig 2:**
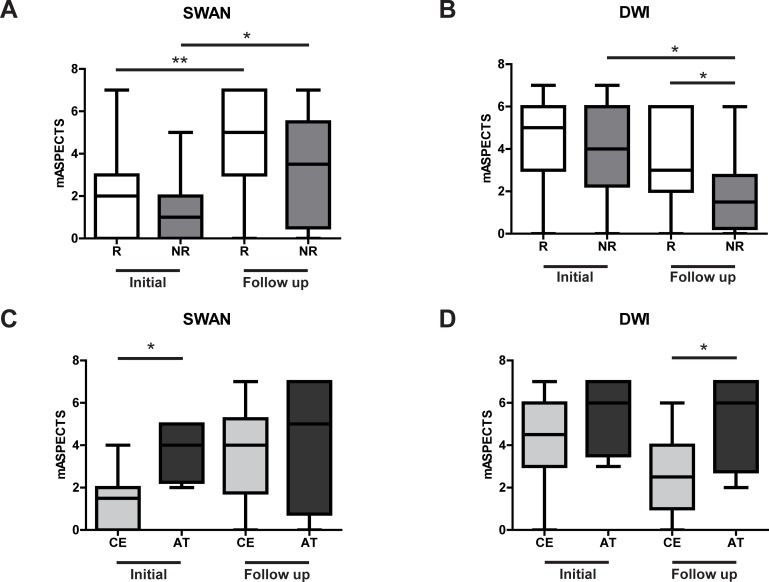
Assessment of the modified Alberta Stroke Program Early CT Score (mASPECTS). **A**. Differences according to recanalization in the T2*-weighted MR angiography (SWAN)-based mASPECTS. In the recanalization (R) and the no recanalization (NR) groups, the initial score was significantly lower than the follow-up score. **B**. Differences according to recanalization in the diffusion-weighted imaging (DWI)-based mASPECTS. The initial score was significantly higher than the follow-up score in the NR group, but initial and follow-up scores in the R group were not significantly different. When comparing follow-up scores, those in the R group were significantly higher than those in the NR group. **C**. Differences in the SWAN-based mASPECTS, by stroke etiology. The initial cardioembolic stroke (CE) group score was significantly lower than the initial atherothrombotic stroke (AT) group score. **D**. Differences by stroke etiology in the DWI-based mASPECTS. The follow-up CE group score was significantly lower than the follow-up AT group score. Mann–Whitney U test: *P < 0.05, **P < 0.01.

The initial median SWAN-based mASPECTSs of 2 (0–3) in the recanalization group and 1 (0–2) in the no recanalization group were significantly lower than the follow-up median SWAN-based mASPECTS, regardless of recanalization: 5 (3–7) in the recanalization group (*P* = 0.001) and 3.5 (0.5–5.5) in the no recanalization group (*P* = 0.045) (Fig 2A). The follow-up median DWI-based mASPECTS of 1.5 (0.25–2.75) was significantly lower than the initial median DWI-based mASPECTS in the no recanalization group of 4 (2.25–6.00) (*P* = 0.018) and the median follow-up DWI-based mASPECTS in the recanalization group of 3 (2.00–6.00) (*P* = 0.037) (Fig 2B). The initial median SWAN-based mASPECTS and the follow-up median DWI-based mASPECTS in the atherothrombotic stroke group of 4 (2.25–5.00) and 6 (2.75–7.00), respectively, were significantly higher than the corresponding scores in the cardioembolic stroke group of 1.5 (0–2.00) and 2.5 (1.00–4.00) (*P* = 0.017 and *P* = 0.042, respectively) (Fig 2C and 2D). In the cardioembolic stroke group, the initial median SWAN-based mASPECTS of 1.5 (0–2.00) was significantly lower than the follow-up median SWAN-based mASPECTS of 4 (1.75–5.25) (*P* = 0.0008). In the cardioembolic stroke group, the initial median DWI-based mASPECTS of 4.5 (3–6) was significantly higher than the follow-up median DWI-based mASPECTS of 2.5 (1–4) (*P* = 0.0064).

**Fig 3:**
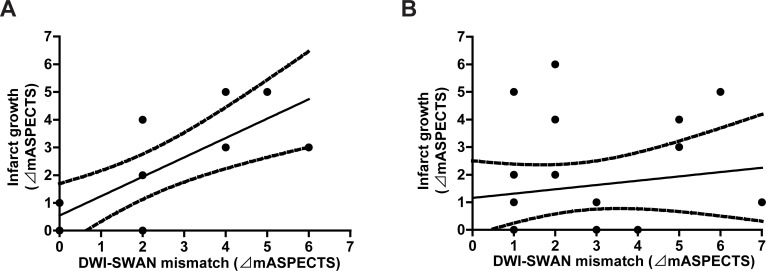
Assessment of the correlation between diffusion-weighted imaging (DWI)-T2*-weighted MR angiography (SWAN) mismatch and infarct growth. DWI-SWAN mismatch was significantly correlated with infarct growth in the no recanalization group (R^2^ = 0.62, *P* < 0.01) (**A**) but not in the recanalization group (**B**).

Of 12 patients showing no recanalization, DWI-SWAN mismatch was significantly correlated with infarct growth (R^2^ = 0.62, *P* = 0.0025) (Fig 3A). In contrast, patients who showed recanalization showed no correlation between mismatch and infarct growth (R^2^ = 0.031, *P* = 0.42) (Fig 3B).

### Assessment of clinical outcome

**Fig 4:**
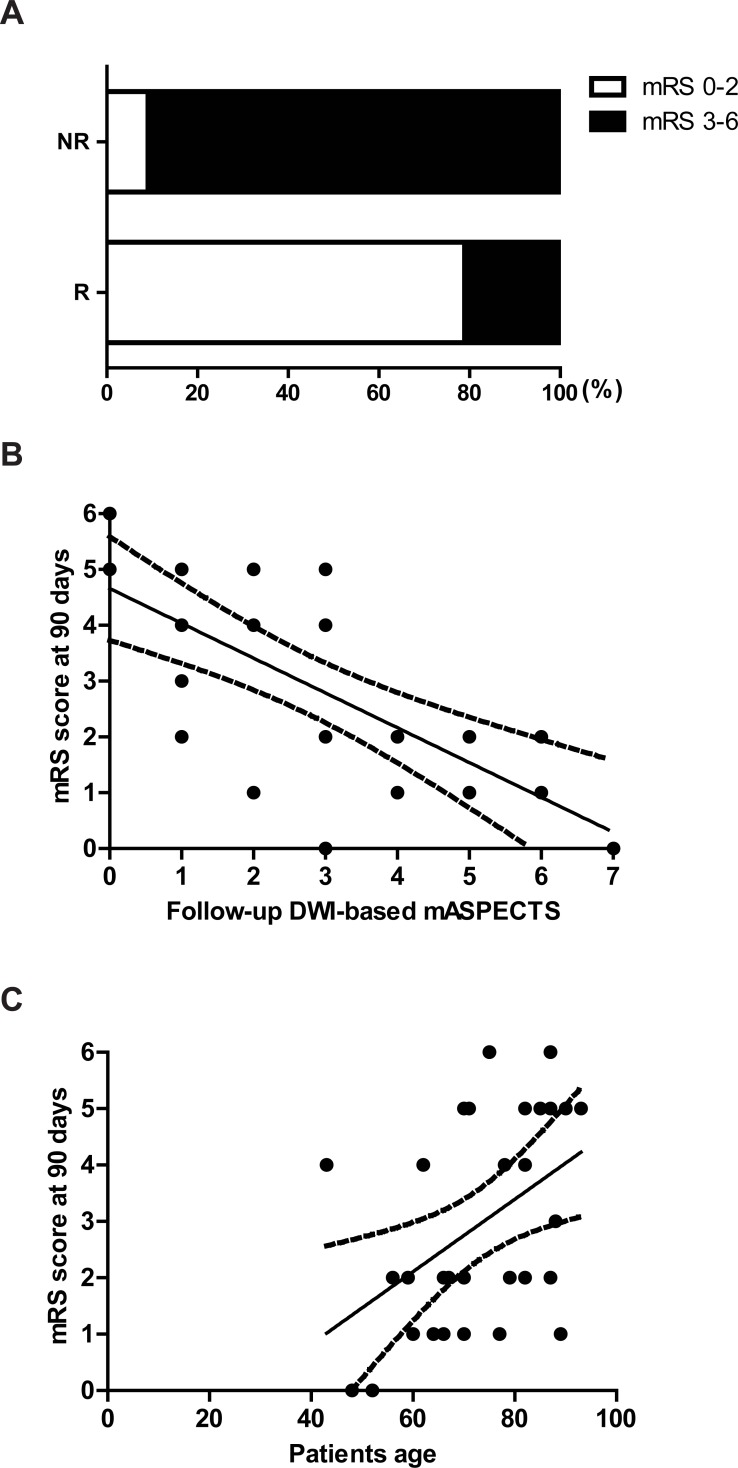
**A. Assessment of clinical outcome with the modified Rankin Scale (mRS) at 90 days after stroke.** The rate of patients with a favorable outcome (mRS scores of 0–2) in the recanalization (R) group was significantly higher than that in the no recanalization (NR) group (78.9% vs. 9.1, *P* < 0.01). **B**. Follow-up diffusion-weighted imaging (DWI)-based mASPECTS was significantly correlated with the mRS score 90 days after stroke (R^2^ = 0.43, *P* < 0.01). **C**. Patient’s age was significantly correlated with the mRS score at 90 days after stroke (R^2^ = 0.24, *P* < 0.01).

With regard to functional outcomes, the median mRS score at 90 days was lower in the recanalization group than in the no recanalization group: 2 (1.00–5.00) vs. 5 (4.00–5.25) (*P* = 0.0002). The favorable outcome rate was significantly higher in the recanalization group than in the no recanalization group (78.9% vs. 9.1%, *P* = 0.004) (Fig 4A). The follow-up DWI-based mASPECTS was significantly correlated with the mRS score at 90 days (R^2^ = 0.43, *P* < 0.0001) (Fig 4B). The patient’s age was significantly correlated with the mRS score at 90 days (R^2^ = 0.24, *P* = 0.006) (Fig 4C).

The variables related to favorable outcome at 90 days after stroke in the univariate analysis were younger age (<80 years), follow-up DWI-based mASPECTS, recanalization, and endovascular treatment. The results of the multivariate logistic regression analysis revealed that follow-up DWI-based mASPECTS [odds ratio 3.15; 95% confidence interval 1.25–15.77; *P* = 0.010] was independently associated with a favorable outcome at 90 days after stroke (Table 2).

**Table 2:** Multivariate logistic regression analysis.

Variables	OR	95% CI	*P* Value
**Younger patients (< 80 years old)**	16.40	0.58–2476.70	0.10
**Follow-up DWI-based mASPECTS**	3.15	1.25–15.77	0.010
**Recanalization**	2.79	0.089–113.09	0.55
**Endovascular treatment**	2.13	0.100–36.06	0.59

OR, odds ratio; CI, confidence interval; DWI, diffusion weighted imaging; mASPECTS, modified Alberta Stroke Program Early CT Score

## Discussion

We learned from this study that (1) the initial SWAN-based mASPECTS was higher in the atherothrombotic stroke group than in the cardioembolic stroke group; (2) DWI-SWAN mismatch predicted infarct growth without acute recanalization; and (3) follow-up DWI-based mASPECTSs were independently associated with a favorable outcome at 90 days after the stroke.

PET showed that misery perfusion caused by intracranial artery occlusion is a condition characterized by an increased brain oxygen extraction fraction (OEF) [14–17]. An increased OEF increases deoxyhemoglobin in the ischemic brain and decreases oxyhemoglobin in venous blood. In an ischemic brain caused by intracranial artery occlusion, a slow-flow state may also contribute to increased deoxyhemoglobin [18]. Decreased OEF indicates irreversible tissue damage [14]. Increased OEF has a risk of progressing to infarction but is salvageable with reperfusion [14]. SWAN is an MRI sequence that is sensitive to paramagnetic substances, such as deoxyhemoglobin. Deoxyhemoglobin is thus a naturally occurring contrast agent for SWAN, specifically termed blood oxygenation level-dependent (BOLD) contrast, which can detect hypointensity in SWAN [19]. In patients with AIS suffering from major vessel occlusion, an increased BOLD effect is believed to reflect an increase in OEF with reduced cerebral blood flow [20]. In fact, the magnetic susceptibility of blood scaled linearly with blood oxygen saturation [21] and asymmetrically prominent cortical veins seen by SWI correspond to reduced levels of oxygen [22]. SWAN after an acute intracranial ICA or M1 occlusion clearly identifies veins with an increased rate of deoxyhemoglobin/oxyhemoglobin as exhibiting hypointensity due to increased OEF-like conditions. Therefore, lesions that SWAN shows have venous hypointensity appear to have viable, salvageable tissue. Perfusion CT or MRI with contrast media differ. Perfusion images show blood flow but do not always indicate viable tissue. Therefore, it has also been reported that the accuracy of SWI-DWI mismatch for predicting favorable outcome was higher than that of perfusion-weighted imaging (PWI)-DWI mismatch [23]. Although PET is one of the gold standard modalities that can indicate viable tissue, these examinations are time-intensive, and the equipment involved is expensive. In patients with AIS, a rapid examination time and immediate reperfusion are required, rendering PET unsuitable. Our method may be applicable to patients with AIS for identifying lesions containing viable tissue without using contrast media as it involves less examination time than perfusion imaging or PET.

Analysis of the DWI-based mASPECTS revealed that acute recanalization prevented infarct growth. Although the initial SWAN-based mASPECTS was significantly lower than the follow-up SWAN-based mASPECTS in both the recanalization and no recanalization groups, the mechanisms likely differ from each other. On the initial MRI, the area with DWI-SWAN mismatch might have imperceptible blood flow from poor leptomeningeal collateral flow. Therefore, the cortical vein or medullary vein was prominent in the area. In the recanalization group, reperfusion during the acute phase reduced the ischemic penumbra, thus potentially decreasing the rate of deoxyhemoglobin to oxyhemoglobin at the time of the follow-up MRI. In contrast, in the no recanalization group, oxygen uptake might have been decreased because of infarction of the affected tissue. However, imperceptible blood flow might have remained in the area. This situation might be similar to that of luxury perfusion. The deoxyhemoglobin-to-oxyhemoglobin conversion rate would not increase enough to show prominent hypointensity of the vein by follow-up MRI. In addition, brain edema caused by infarction might affect higher follow-up SWAN-based mASPECTS in the no recanalization group compared with the initial SWAN-based mASPECTS [24]. Both of these mechanisms would decrease a lesion’s cortical venous hypointensity in follow-up SWAN. With regard to stroke etiology, susceptibility vessel signs picked up on SWAN appear useful for distinguishing between cardioembolic and atherosclerotic in situ stenosis/occlusion [25]. Therefore, the initial SWAN-based mASPECTS might help when considering stroke etiology. We found that there was a significantly higher initial SWAN-based mASPECTS with atherothrombotic stroke than with cardioembolic stroke. In atherothrombotic stroke, the major artery becomes gradually constricted such that collateral flow could develop [26]. Therefore, even if the artery is occluded, the volume of ischemic penumbra would be small and the mASPECTS of the initial SWAN would be higher because of good collateral flow.

The DWI-SWAN lesion mismatch seen on the initial MRI in our study was strongly correlated with new infarct lesion growth in patients with no recanalization. In contrast, when recanalization was achieved during the acute phase, there was no correlation between the DWI-SWAN lesion mismatch and infarct lesion growth. Kaya et al. reported that the lesion volume of multiple hypointense vessels on T2* MRI at 3 T was correlated with infarct volume at 72 h and with PWI lesion volumes with a prolonged mean transit time, delayed time to peak, decreased relative cerebral blood flow, and increased relative cerebral blood volume [7]. Luo et al. reported that SWI-DWI mismatch was positively correlated with the DWI-mean transit time mismatches [27]. Kao et al. reported that the SWI-DWI mismatch predicted infarct growth without reperfusion and that SWI could be used to assess penumbra [6]. Lou et al. reported that the presence of an SWI-DWI mismatch predicted a favorable outcome by reperfusion or recanalization [23]. In our study, lesions with a DWI-SWAN mismatch during the hyperacute phase shown by MRI were considered for salvaging lesion tissue by acute recanalization and for identifying ischemic penumbra-like lesions. We believe that the DWI-SWAN mismatch is a positive indicator when considering recanalization treatment for patients with AIS. The results of the DAWN trial and DEFUSE 3 required assessment of residual ischemic penumbra [3, 4]. Although an MRA-DWI mismatch and PWI-DWI mismatch would be useful for assessing ischemic penumbra in the hyperacute phase, especially <6 h from onset [28, 29], a DWI-SWAN mismatch might provide helpful information concerning residual ischemic penumbra during the acute phase (i.e., >6 h from onset).

Follow-up DWI-based mASPECTS were significantly correlated with the mRS score 90 days after stroke, and follow-up DWI-based mASPECTS was independently associated with favorable outcomes 90 days after stroke. There was no correlation between the follow-up DWI-based mASPECTS and the onset-to-recanalization time in the recanalization group. Embolization into new territory might affect lower follow-up DWI-based mASPECTS. The prevention of such embolization would be needed [30].

Although age < 80 years was not independently associated with a favorable outcome at 90 days, the patient’s age was significantly correlated with the mRS score 90 days after stroke. Patient’s age also affected the clinical outcome. This finding was consistent with previous reports [31, 32]. Aging was reported to not only reduce the number of stem cells but also stem cell function, including proliferative activity and trophic factor secretion from stem cells [33–35]. Also, the capacity of neurogenesis in older rats was reported to be lower than that in young adult rats after ischemic stroke [36]. Not only the higher burden of comorbidities and complications after stroke but also the lower regenerative capacity in elderly patients would affect decreased functional recovery after stroke compared with that of young patients [32].

Our study has several limitations. First, there is no protocol concerning oxygen inhalation. Oxygen inhalation decreases the deoxyhemoglobin/oxyhemoglobin ratio. Therefore, the hypointensity seen by SWAN can easily disappear. Because some of our patients were on oxygen inhalation, some affected lesions might have been underestimated. Second, we could not compare lesions of DWI-SWAN mismatch with ischemic penumbra as indicated by PWI, single photon emission CT, or PET. Third, we did not rate basal ganglia complex (I, C, L, IC) infarction by mASPECTS because it was difficult to evaluate the deep drainage pattern in the basal ganglia complex [10]. Fourth, SWAN provides relatively poorer contrast and spatial resolution than MRA or CTA using contrast media, so it is difficult to evaluate the collateral supply with SWAN alone. Fifth, we could not perform a quantitative evaluation of the hypointense areas in medullary or cortical veins seen with SWAN. In the clinical setting, it is most important to evaluate an ischemic penumbra rapidly. ASPECTS-DWI is a well-known means to evaluate infarction areas. Thus, we used mAPECTS to evaluate the hypointense areas of medullary or cortical veins seen with SWAN [6, 10, 27]. Furthermore, this method would be useful for assessing areas where reperfusion is needed for endovascular therapy. Quantitative evaluation of hypointense areas in medullary or cortical veins by SWAN would provide more reliable information. Although Lou et al. evaluated the hypointense area in medullary or cortical veins by measuring the pixel number [23], it might be necessary to comprehensively take the susceptibility value, the number of prominent hypointensity vessels, and the prominent hypointensity vessel size into consideration to evaluate perfusion insufficiency. New methods that are simple and take less time need to be developed. Sixth, the sample size was small in our study, which might have affected the statistical analysis. Therefore, further investigation on a larger sample size is required.

In conclusion, our study suggests that the DWI-SWAN mismatch in 1.5-T MRI can identify ischemic penumbra-like lesions in patients with AIS and acute major vessel occlusion. Additionally, this mismatch may indicate the extent to which lesions without acute recanalization progress to infarction. SWAN would also be of some help to diagnose the stroke's etiology. Assessment of ischemic penumbra-like lesions from the venous side by SWAN is highly useful, with no need for contrast media.

## References

[1] Goyal M, Menon BK, van Zwam WH, Dippel DW, Mitchell PJ, Demchuk AM, et al Endovascular thrombectomy after large-vessel ischaemic stroke: a meta-analysis of individual patient data from five randomised trials. Lancet. 2016;387(10029):1723–31. Epub 2016/02/24. doi: 10.1016/S0140-6736(16)00163-X . 26898852 10.1016/S0140-6736(16)00163-X

[2] Saver JL, Goyal M, van der Lugt A, Menon BK, Majoie CB, Dippel DW, et al Time to Treatment With Endovascular Thrombectomy and Outcomes From Ischemic Stroke: A Meta-analysis. JAMA. 2016;316(12):1279–88. Epub 2016/09/28. doi: 10.1001/jama.2016.13647 . 27673305 10.1001/jama.2016.13647

[3] Nogueira RG, Jadhav AP, Haussen DC, Bonafe A, Budzik RF, Bhuva P, et al Thrombectomy 6 to 24 Hours after Stroke with a Mismatch between Deficit and Infarct. N Engl J Med. 2018;378(1):11–21. Epub 2017/11/14. doi: 10.1056/NEJMoa1706442 . 29129157 10.1056/NEJMoa1706442

[4] Albers GW, Marks MP, Kemp S, Christensen S, Tsai JP, Ortega-Gutierrez S, et al Thrombectomy for Stroke at 6 to 16 Hours with Selection by Perfusion Imaging. N Engl J Med. 2018 Epub 2018/01/25. doi: 10.1056/NEJMoa1713973 . 29364767 10.1056/NEJMoa1713973 PMC6590673

[5] Hartkamp NS, van Osch MJ, Kappelle J, Bokkers RP. Arterial spin labeling magnetic resonance perfusion imaging in cerebral ischemia. Curr Opin Neurol. 2014;27(1):42–53. Epub 2013/12/05. doi: 10.1097/WCO.0000000000000051 . 24300794 10.1097/WCO.0000000000000051

[6] Kao HW, Tsai FY, Hasso AN. Predicting stroke evolution: comparison of susceptibility-weighted MR imaging with MR perfusion. Eur Radiol. 2012;22(7):1397–403. Epub 2012/02/11. doi: 10.1007/s00330-012-2387-4 . 22322311 10.1007/s00330-012-2387-4

[7] Kaya D, Dincer A, Yildiz ME, Cizmeli MO, Erzen C. Acute ischemic infarction defined by a region of multiple hypointense vessels on gradient-echo T2* MR imaging at 3T. AJNR Am J Neuroradiol. 2009;30(6):1227–32. Epub 2009/04/07. doi: 10.3174/ajnr.A1537 . 19346312 10.3174/ajnr.A1537 PMC7051320

[8] Park MG, Yang TI, Oh SJ, Baik SK, Kang YH, Park KP. Multiple hypointense vessels on susceptibility-weighted imaging in acute ischemic stroke: surrogate marker of oxygen extraction fraction in penumbra? Cerebrovasc Dis. 2014;38(4):254–61. Epub 2014/11/18. doi: 10.1159/000367709 . 25401484 10.1159/000367709

[9] Chen CY, Chen CI, Tsai FY, Tsai PH, Chan WP. Prominent vessel sign on susceptibility-weighted imaging in acute stroke: prediction of infarct growth and clinical outcome. PLoS One. 2015;10(6):e0131118 Epub 2015/06/26. doi: 10.1371/journal.pone.0131118 ; PubMed Central PMCID: PMCPMC4481350. 26110628 10.1371/journal.pone.0131118 PMC4481350

[10] Cheng B, Schroder N, Forkert ND, Ludewig P, Kemmling A, Magnus T, et al Hypointense Vessels Detected by Susceptibility-Weighted Imaging Identifies Tissue at Risk of Infarction in Anterior Circulation Stroke. J Neuroimaging. 2016 Epub 2016/12/22. doi: 10.1111/jon.12417 . 28000975 10.1111/jon.12417

[11] Kawano H, Hirano T, Inatomi Y, Terasaki T, Yonehara T, Uchino M. Presence of deep white matter lesions on diffusion-weighted imaging is a negative predictor of early dramatic improvement after intravenous tissue plasminogen activator thrombolysis. Cerebrovasc Dis. 2010;30(3):230–6. Epub 2010/07/08. doi: 10.1159/000317183 . 20606424 10.1159/000317183

[12] Kawano H, Hirano T, Nakajima M, Inatomi Y, Yonehara T, Uchino M. Modified ASPECTS for DWI including deep white matter lesions predicts subsequent intracranial hemorrhage. J Neurol. 2012;259(10):2045–52. Epub 2012/02/22. doi: 10.1007/s00415-012-6446-1 ; PubMed Central PMCID: PMCPmc3464370. 22349869 10.1007/s00415-012-6446-1 PMC3464370

[13] Maclure M, Willett WC. Misinterpretation and misuse of the kappa statistic. Am J Epidemiol. 1987;126(2):161–9. Epub 1987/08/01. . 3300279 10.1093/aje/126.2.161

[14] Heiss WD. Ischemic penumbra: evidence from functional imaging in man. J Cereb Blood Flow Metab. 2000;20(9):1276–93. Epub 2000/09/20. doi: 10.1097/00004647-200009000-00002 . 10994849 10.1097/00004647-200009000-00002

[15] Baron JC, Bousser MG, Rey A, Guillard A, Comar D, Castaigne P. Reversal of focal "misery-perfusion syndrome" by extra-intracranial arterial bypass in hemodynamic cerebral ischemia. A case study with 15O positron emission tomography. Stroke. 1981;12(4):454–9. Epub 1981/07/01. . 6976022 10.1161/01.str.12.4.454

[16] Heiss WD, Graf R, Lottgen J, Ohta K, Fujita T, Wagner R, et al Repeat positron emission tomographic studies in transient middle cerebral artery occlusion in cats: residual perfusion and efficacy of postischemic reperfusion. J Cereb Blood Flow Metab. 1997;17(4):388–400. Epub 1997/04/01. doi: 10.1097/00004647-199704000-00004 . 9143221 10.1097/00004647-199704000-00004

[17] Lee JM, Vo KD, An H, Celik A, Lee Y, Hsu CY, et al Magnetic resonance cerebral metabolic rate of oxygen utilization in hyperacute stroke patients. Ann Neurol. 2003;53(2):227–32. Epub 2003/01/31. doi: 10.1002/ana.10433 . 12557290 10.1002/ana.10433

[18] Kennan RP, Zhong J, Gore JC. Intravascular susceptibility contrast mechanisms in tissues. Magn Reson Med. 1994;31(1):9–21. Epub 1994/01/01. . 8121277 10.1002/mrm.1910310103

[19] Ogawa S, Lee TM, Kay AR, Tank DW. Brain magnetic resonance imaging with contrast dependent on blood oxygenation. Proc Natl Acad Sci U S A. 1990;87(24):9868–72. Epub 1990/12/01. ; PubMed Central PMCID: PMCPMC55275. 2124706 10.1073/pnas.87.24.9868 PMC55275

[20] Morita N, Harada M, Uno M, Matsubara S, Matsuda T, Nagahiro S, et al Ischemic findings of T2*-weighted 3-tesla MRI in acute stroke patients. Cerebrovasc Dis. 2008;26(4):367–75. Epub 2008/08/30. doi: 10.1159/000151640 . 18728364 10.1159/000151640

[21] Langham MC, Floyd TF, Mohler ER 3rd, Magland JF, Wehrli FW. Evaluation of cuff-induced ischemia in the lower extremity by magnetic resonance oximetry. J Am Coll Cardiol. 2010;55(6):598–606. Epub 2010/02/16. doi: 10.1016/j.jacc.2009.08.068 ; PubMed Central PMCID: PMCPMC2833093. 20152564 10.1016/j.jacc.2009.08.068 PMC2833093

[22] Xia S, Utriainen D, Tang J, Kou Z, Zheng G, Wang X, et al Decreased oxygen saturation in asymmetrically prominent cortical veins in patients with cerebral ischemic stroke. Magn Reson Imaging. 2014;32(10):1272–6. Epub 2014/08/19. doi: 10.1016/j.mri.2014.08.012 . 25131626 10.1016/j.mri.2014.08.012

[23] Lou M, Chen Z, Wan J, Hu H, Cai X, Shi Z, et al Susceptibility-diffusion mismatch predicts thrombolytic outcomes: a retrospective cohort study. AJNR Am J Neuroradiol. 2014;35(11):2061–7. Epub 2014/07/12. doi: 10.3174/ajnr.A4017 . 25012670 10.3174/ajnr.A4017 PMC7965182

[24] Baik SK, Choi W, Oh SJ, Park KP, Park MG, Yang TI, et al Change in cortical vessel signs on susceptibility-weighted images after full recanalization in hyperacute ischemic stroke. Cerebrovasc Dis. 2012;34(3):206–12. Epub 2012/09/26. doi: 10.1159/000342148 . 23006622 10.1159/000342148

[25] Horie N, Tateishi Y, Morikawa M, Morofuji Y, Hayashi K, Izumo T, et al Acute stroke with major intracranial vessel occlusion: Characteristics of cardioembolism and atherosclerosis-related in situ stenosis/occlusion. J Clin Neurosci. 2016;32:24–9. Epub 2016/08/11. doi: 10.1016/j.jocn.2015.12.043 . 27506779 10.1016/j.jocn.2015.12.043

[26] Toyoda K, Minematsu K, Yamaguchi T. Long-term changes in cerebral blood flow according to different types of ischemic stroke. J Neurol Sci. 1994;121(2):222–8. Epub 1994/02/01. . 8158219 10.1016/0022-510x(94)90356-5

[27] Luo S, Yang L, Wang L. Comparison of susceptibility-weighted and perfusion-weighted magnetic resonance imaging in the detection of penumbra in acute ischemic stroke. J Neuroradiol. 2015;42(5):255–60. Epub 2014/12/03. doi: 10.1016/j.neurad.2014.07.002 . 25451668 10.1016/j.neurad.2014.07.002

[28] Parsons MW, Christensen S, McElduff P, Levi CR, Butcher KS, De Silva DA, et al Pretreatment diffusion- and perfusion-MR lesion volumes have a crucial influence on clinical response to stroke thrombolysis. J Cereb Blood Flow Metab. 2010;30(6):1214–25. Epub 2010/01/21. doi: 10.1038/jcbfm.2010.3 ; PubMed Central PMCID: PMCPMC2949206. 20087363 10.1038/jcbfm.2010.3 PMC2949206

[29] Lansberg MG, Thijs VN, Bammer R, Olivot JM, Marks MP, Wechsler LR, et al The MRA-DWI mismatch identifies patients with stroke who are likely to benefit from reperfusion. Stroke. 2008;39(9):2491–6. Epub 2008/07/19. doi: 10.1161/STROKEAHA.107.508572 ; PubMed Central PMCID: PMCPMC3985810. 18635861 10.1161/STROKEAHA.107.508572 PMC3985810

[30] Stampfl S, Pfaff J, Herweh C, Pham M, Schieber S, Ringleb PA, et al Combined proximal balloon occlusion and distal aspiration: a new approach to prevent distal embolization during neurothrombectomy. J Neurointerv Surg. 2017;9(4):346–51. Epub 2016/04/09. doi: 10.1136/neurintsurg-2015-012208 . 27056920 10.1136/neurintsurg-2015-012208

[31] Shi ZS, Liebeskind DS, Xiang B, Ge SG, Feng L, Albers GW, et al Predictors of functional dependence despite successful revascularization in large-vessel occlusion strokes. Stroke. 2014;45(7):1977–84. Epub 2014/05/31. doi: 10.1161/STROKEAHA.114.005603 ; PubMed Central PMCID: PMCPMC4160889. 24876082 10.1161/STROKEAHA.114.005603 PMC4160889

[32] Sanossian N, Apibunyopas KC, Liebeskind DS, Starkman S, Burgos AM, Conwit R, et al Characteristics and Outcomes of Very Elderly Enrolled in a Prehospital Stroke Research Study. Stroke. 2016 Epub 2016/09/30. doi: 10.1161/strokeaha.116.013318 . 27679533 10.1161/STROKEAHA.116.013318

[33] Khan M, Mohsin S, Khan SN, Riazuddin S. Repair of senescent myocardium by mesenchymal stem cells is dependent on the age of donor mice. J Cell Mol Med. 2011;15(7):1515–27. Epub 2010/01/01. doi: 10.1111/j.1582-4934.2009.00998.x ; PubMed Central PMCID: PMCPMC3823196. 20041970 10.1111/j.1582-4934.2009.00998.x PMC3823196

[34] Thijssen DH, Vos JB, Verseyden C, van Zonneveld AJ, Smits P, Sweep FC, et al Haematopoietic stem cells and endothelial progenitor cells in healthy men: effect of aging and training. Aging Cell. 2006;5(6):495–503. Epub 2006/11/04. doi: 10.1111/j.1474-9726.2006.00242.x . 17081158 10.1111/j.1474-9726.2006.00242.x

[35] Yamaguchi S, Horie N, Satoh K, Ishikawa T, Mori T, Maeda H, et al Age of donor of human mesenchymal stem cells affects structural and functional recovery after cell therapy following ischaemic stroke. J Cereb Blood Flow Metab. 2017:271678X17731964. Epub 2017/09/16. doi: 10.1177/0271678x17731964 . 28914133 10.1177/0271678X17731964 PMC6434451

[36] Chen Y, Sun FY. Age-related decrease of striatal neurogenesis is associated with apoptosis of neural precursors and newborn neurons in rat brain after ischemia. Brain Res. 2007;1166:9–19. Epub 2007/07/31. doi: 10.1016/j.brainres.2007.06.043 . 17662700 10.1016/j.brainres.2007.06.043

